# Left ventricular end-diastolic dimension and septal e′ are predictors of cardiac index at rest, while tricuspid annular plane systolic excursion is a predictor of peak oxygen uptake in patients with pulmonary hypertension

**DOI:** 10.1007/s00380-017-1086-0

**Published:** 2017-11-15

**Authors:** Yoshihisa Nakano, Naoki Okumura, Shiro Adachi, Shigetake Shimokata, Fumitaka Tajima, Yoshihiro Kamimura, Toyoaki Murohara, Takahisa Kondo

**Affiliations:** 1 Department of Cardiology, Nagoya University Graduate School of Medicine, Nagoya, Japan; 2 Department of Advanced Medicine in Cardiopulmonary Disease, Nagoya University Graduate School of Medicine, 65 Tsurumai-cho, Shouwa-ku, Nagoya, 466-8560 Japan

**Keywords:** Pulmonary hypertension, Echocardiography, Cardiac index, Peak oxygen uptake

## Abstract

Little is known regarding a correlation of hemodynamics at rest or exercise capacity with echocardiographic parameters in patients with pulmonary hypertension (PH). To clarify these potential correlations, we performed transthoracic echocardiography, right heart catheterization, and cardiopulmonary exercise testing in 53 patients with pulmonary arterial hypertension (PAH) and chronic thromboembolic pulmonary hypertension (CTEPH). Left ventricular end-diastolic dimension (LVDd), early diastolic velocity of the septal mitral annulus (septal e′), tricuspid regurgitation peak gradient (TRPG), and tricuspid annular plane systolic excursion (TAPSE) were significantly correlated with cardiac index (LVDd; *r* = 0.477, *P* < 0.001, septal e′; *r* = 0.463, *P* = 0.001, TRPG; *r* = − 0.455, *P* = 0.001 and TAPSE; *r* = 0.406, *P* = 0.003, respectively). Multiple regression analysis revealed that LVDd and septal e′ were significantly associated with cardiac index (CI) and stroke volume index at rest. Among the exercise capacity markers evaluated, TAPSE, TRPG, and LVDd were significantly correlated with peak oxygen uptake (TAPSE; *r* = 0.534, *P* < 0.001, TRPG; *r* = − 0.466, *P* = 0.001 and LVDd; *r* = 0.411, *P* = 0.002, respectively). Multiple regression analysis showed that TAPSE was significantly associated with peak oxygen uptake (VO_2_). In PAH and CTEPH patients, LVDd and septal e′ were significantly associated with CI at rest, whereas TAPSE was significantly associated with peak VO_2_. Echocardiographic parameters may predict the prognostic factors of PAH and CTEPH patients.

**Electronic supplementary material:**

The online version of this article (10.1007/s00380-017-1086-0) contains supplementary material, which is available to authorized users.

## Introduction

Several specific therapies have been introduced for the treatment of pulmonary arterial hypertension (PAH) and chronic thromboembolic pulmonary hypertension (CTEPH), and patient conditions have been assessed repeatedly in detail. [1–6] Noninvasive techniques such as echocardiography and MRI are used for assessing the severity of PAH or CTEPH [7–14]. Echocardiography is a particularly convenient method in routine medical care, and measures several parameters helpful for evaluating cardiac function [15, 16]. In particular, in the assessment of right cardiac function, the tricuspid regurgitation peak gradient (TRPG) is useful for estimating the pulmonary artery pressure (PAP), whereas tricuspid annular plane systolic excursion (TAPSE), a well-known prognostic factor of PAH, is a measure of the systolic function of the right ventricle (RV) [17–19]. In addition, echocardiography and MRI have proven to be valuable tools in estimating right-to-left ventricular interaction and diastolic dysfunction of the left ventricle (LV) associated with deleterious increases in RV pressure [20–22].

Cardiac index (CI), which is obtained during right heart catheterization, and peak oxygen uptake (VO_2_), which is assessed through cardiopulmonary exercise testing (CPET), are established prognostic factors of PAH [23]. However, details of the correlation between these invasively acquired prognostic factors and noninvasive echocardiographic parameters are still not enough in patients with PAH and CTEPH. Accordingly, we investigated the potential relationship among hemodynamics at rest, exercise capacity, and the echocardiographic parameters routinely measured at rest in patients with PAH and CTEPH.

## Materials and methods

### Patients and study design

**Fig. 1:**
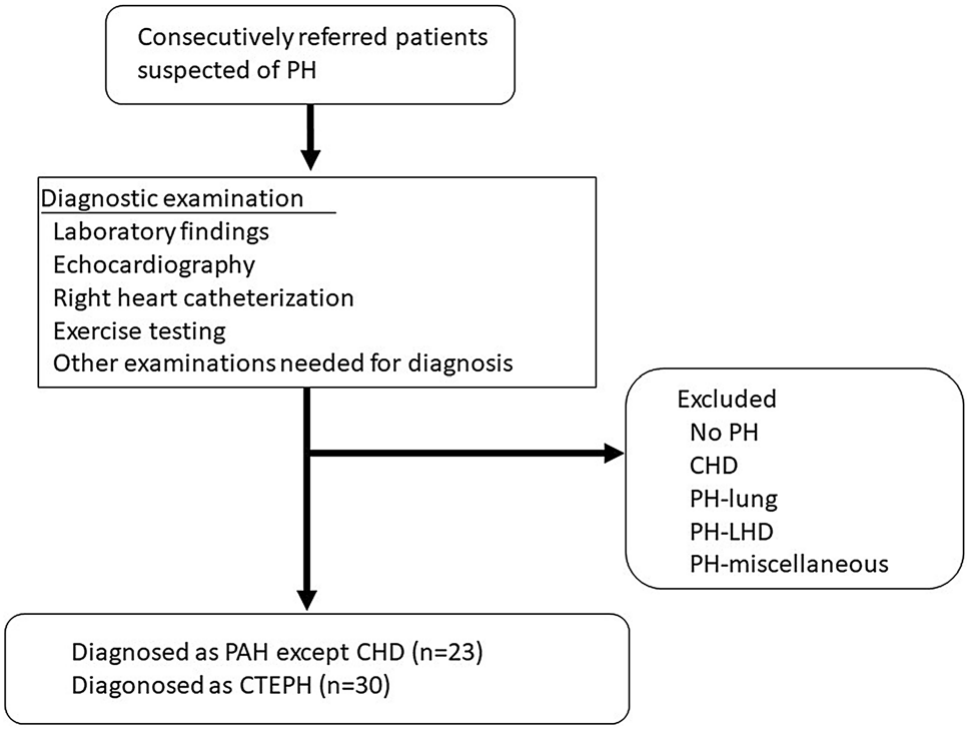
Flowchart of the inclusion process. *PH* pulmonary hypertension; *PAH* pulmonary arterial hypertension; *CTEPH* chronic thromboembolic pulmonary hypertension; *CHD* congenital heart disease; *PH-lung* pulmonary hypertension associated with lung disease; *PH-LHD* pulmonary hypertension associated with left heart disease

We retrospectively enrolled 53 consecutive patients with PAH and CTEPH who were referred to our institution for the first time between April 1, 2012 and May 31, 2016 (Fig. 1). All patients underwent transthoracic echocardiography, right heart catheterization, and CPET. Patients were diagnosed as having pulmonary hypertension (PH) when right heart catheterization demonstrated a mean PAP of ≥ 25 mmHg and a pulmonary arterial wedge pressure (PAWP) of ≤ 15 mmHg. Patients with obstructive or restrictive lung disease, left heart disease, or congenital heart disease were excluded. This study was approved by the human research ethics committees of Nagoya University Hospital (no. 2016-0275).

### Hemodynamic studies

All patients underwent right heart catheterization via right internal jugular vein with a 6-French Thermodilution catheter (Goodman Co. LTD, Nagoya, Japan) to obtain the pulmonary arterial wedge pressure (PAWP), PAP, RV, and right atrial (RA) pressures. Mixed venous oxygen saturation and arterial oxygen saturation were measured in blood taken from each main PA and radial artery. Cardiac output was calculated by indirect Fick method and estimated VO_2_ values were calculated by LaFarge equation: males, VO_2_ (mL/min/m^2^) = [138.1 − (11.49 × log_e_age) + (0.378 × HR)] × BSA; females, VO_2_ (mL/min/m^2^) = [138.1 − (17.04 × log_e_age) + (0.378 × HR)] × BSA. Pulmonary vascular resistance (PVR) was calculated using the standard formula: PVR = 80 × (mean PAP − mean PAWP)/cardiac output.

### Cardiopulmonary exercise testing

All 53 patients underwent exercise testing at a progressively increasing work rate to maximum tolerance on a cycle ergometer. The test was performed according to the American Thoracic Society guidelines [24]. Specifically, 3 min of warm-up was performed with a 10-W loading, followed by a 10-W/min ramp-incremental protocol. Carbon dioxide output (VCO_2_), VO_2_, and minute ventilation (VE) were measured continuously on a breath-by-breath basis using an Ergospirometry Oxycon Pro (Carefusion Germany, 234, GmbH, Hochberg, Germany).

### Echocardiography

Two-dimensional, M-mode, Doppler echocardiographic images were acquired (iE33, Philips Healthcare, Eindhoven, The Netherlands) and examined according to the guidelines of the American Society of Echocardiography [25, 26]. Patients were examined in the left decubitus position through parasternal long-axis, short-axis, and apical views. The left ventricular internal dimensions at end diastole (LVDd) and end systole (LVDs) were measured in the standard parasternal long-axis view at the level of the mitral valve leaflet tips obtained from two-dimensional echocardiographic images. End diastole was defined as the frame in the cardiac cycle in which the respective LV dimension is the largest. The peak velocity of tricuspid regurgitation was measured by using continuous-wave Doppler echocardiography. Doppler imaging of the mitral valve was performed in the apical four-chamber view. Peak E and peak A wave velocities were obtained from the mitral inflow velocity image. TAPSE was measured using M-mode echocardiography with the cursor placed at the tricuspid lateral annulus in the apical four-chamber view. TAPSE is defined by the total excursion of the tricuspid annulus from its highest position after atrial ascent to the peak descent during ventricular systole. Tissue Doppler echocardiography was performed in the apical four-chamber view, with the tissue sampling volume located at the septal side of the mitral annulus, and the early diastolic velocity wave (e′) was measured.

### Statistical analysis

Categorical variables are presented as numbers and percentages (%). Continuous variables are presented as mean ± standard deviation or medians. Pearson correlation was used to assess the strength of the relationship between two variables. We performed stepwise multiple regression analysis to evaluate factors that affected CI, stroke volume index (SVI), and peak VO_2_. Variables including clinical and laboratory findings, and hemodynamic and echocardiographic parameters were selected on the basis of a significant univariate relation (*P* < 0.05). A *P* value of < 0.05 was considered to be statistically significant. All statistical analyses were conducted by using the SPSS statistical software program (SPSS version 18.0 for Windows, SPSS Inc., Chicago, IL, USA).

## Results

### Patient characteristics

Table 1 shows patient characteristics. The mean age of the patients was 53.7 years, and 16 (30%) were men. Among 53 patients, 23 patients had PAH and 30 had CTEPH. Of these 53 patients, 12 (23%) were already receiving oral PH-specific drug therapy prescribed in a referral hospital. The remaining 41 (77%) patients were treatment naive. 19 (36%) were in WHO functional class III/IV.

**Table 1:** Patient characteristics

Number of patients	53
I/HPAH	12
POPH	8
CTD-PAH	3
CTEPH	30
Age (years)	53.7 ± 15.2
Male	16
Height (cm)	159.3 ± 10.0
Weight (kg)	61.1 ± 16.0
Body mass index (kg m^−2^)	24.0 ± 5.9
Body surface area (m^2^)	1.6 ± 0.2
WHO functional class	
1/2/3/4	0/34/15/4
6MWD (m)	380 ± 98
Monotherapy	
ERA	4
PDE-5	4
Combination therapy	
ERA + PDE-5	4
Plasma BNP (pg/mL)	102.0 (36.3–276.3)

Data presented as mean ± SD, median (interquartile range) or *n*

*I/HPAH* idiopathic/heritable pulmonary arterial hypertension; *POPH* portopulmonary hypertension; *CTD-PAH* connective tissue disease-associated pulmonary arterial hypertension; *CTEPH* chronic thromboembolic pulmonary hypertension; *WHO* World Health Organization; *6MWD* six-minute walk distance; *ERA* endothelin receptor antagonist; *PDE-5* phosphodiesterase-5 inhibitor; *BNP* brain natriuretic peptide

### Hemodynamics at rest and echocardiography

The mean TRPG and TAPSE in 53 patients were 74.1 mmHg and 17.3 mm, respectively, highly indicative of severe PH (Table 2). As patients who had left heart disease had been excluded from the enrollment, the LV internal dimension, LV wall thickness, and left atrial dimension (LAD) of our patients were all within normal limits. In our PH patients, the septal e′ was decreased and E/A ratio was reduced, both suggesting impaired left ventricular relaxation. Meanwhile, overall hemodynamic data from our patient population suggested severe PH with low cardiac output.

**Table 2:** Baseline measured variables

Number of patients	53
Hemodynamics	
PAWP (mmHg)	9.5 ± 3.7
Mean ABP (mmHg)	93.1 ± 13.6
Mean RAP (mmHg)	6.7 ± 4.3
Systolic PAP (mmHg)	80.0 ± 16.5
Diastolic PAP (mmHg)	28.9 ± 8.9
Mean PAP (mmHg)	47.8 ± 10.4
Heart rate (bpm)	76.5 ± 15.1
SvO_2_ (%)	63.5 ± 9.7
SaO_2_ (%)	91.9 ± 4.9
CI (l/min/m^2^)	2.1 ± 0.7
SVI (l/m^2^)	28.7 ± 10.5
PVR (Wood Unit)	12.6 ± 6.8
Cardiopulmonary exercise test	
Peak VO_2_ (ml/kg/min)	13.8 ± 4.1
O_2_ pulse	6.5 ± 2.2
VE/VCO_2_ slope	52.9 ± 17.7
Peak RER	1.0 ± 0.1
Echocardiography	
LVDd (mm)	40.1 ± 6.0
LVDs (mm)	25.3 ± 4.5
IVSd (mm)	8.3 ± 1.3
LVPWd (mm)	8.4 ± 1.3
LAD (mm)	32.4 ± 5.9
TRPG (mmHg)	74.1 ± 23.4
TR grade	
I/II/III/IV	9/17/15/11
TAPSE (mm)	17.3 ± 4.9
E velocity (cm/s)	56.8 ± 19.0
A velocity (cm/s)	69.5 ± 18.0
E/A	0.9 ± 0.4
DcT (ms)	224.8 ± 81.4
Septal e′ (cm/s)	5.2 ± 2.0
E/e′	12.2 ± 5.4

Data are presented as mean ± SD or *n*

*PAWP* pulmonary artery wedge pressure; *ABP* arterial blood pressure; *RAP* right atrial pressure; *PAP* pulmonary artery pressure; *SvO*_*2*_ mixed venous oxygen saturation; *SaO*_*2*_ arterial oxygen saturation; *CI* cardiac index; *SVI* stroke volume index; *PVR* pulmonary vascular resistance; *HR* heart rate; *VO*_*2*_ oxygen uptake; *VE* minute ventilation; *VCO*_*2*_ carbon dioxide production; *RER* respiratory exchange ratio; *LVDd* left ventricular end-diastolic dimension; *LVSd* left ventricular end-systolic dimension; *IVSd* interventricular septal distance; *LVPWd* left ventricular posterior wall distance; *LAD* left atrial dimension; *TRPG* pressure gradient of tricuspid regurgitation; *TR* tricuspid regurgitation; *TAPSE* tricuspid annular plane systolic excursion; *DcT* deceleration time; *e′* early diastolic velocity of mitral annulus

**Fig. 2:**
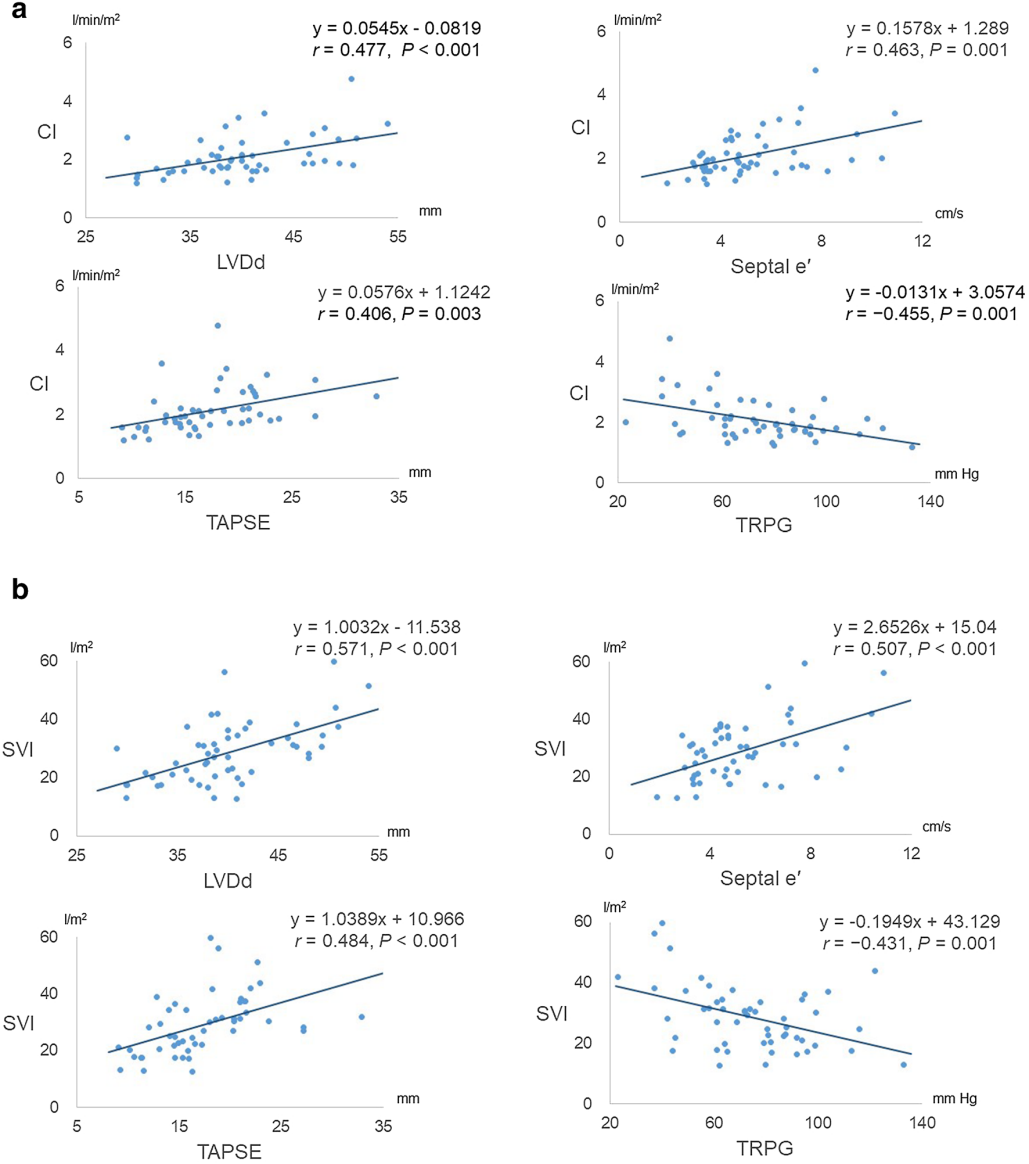
**a** Relation of cardiac index (CI) to left ventricular end-diastolic dimension (LVDd), early diastolic velocity of the septal mitral annulus (septal e′), tricuspid annular plane systolic excursion (TAPSE), and pressure gradient of tricuspid regurgitation (TRPG). **b** Relation of stroke volume index (SVI) to LVDd, e′, TAPSE, and TRPG

Correlations of echocardiographic parameters and hemodynamics at rest are shown in Fig. 2 and Supplement 3. TRPG was significantly and positively correlated with mean PAP, while LVDd and TAPSE were significantly and negatively correlated with mean PAP. LVDd, septal e′, and TAPSE were significantly and positively correlated with CI and SVI, while TRPG was significantly and negatively correlated with CI and SVI.

Then, stepwise multiple regression analysis was performed to analyze which factors influenced CI, a well-known prognostic marker for PAH, in our patients. Among the variables including clinical and laboratory findings, hemodynamic and echocardiographic parameters, the independent variables used in this analysis, were selected on the basis of a significance of univariate analysis: age, brain natriuretic peptide (BNP), mean PAP, E, septal e′, LVDd, TAPSE, and TRPG. We also included heart rate (HR) as an independent variable for clinical importance. SvO_2_ was excluded from the analysis because it was included in the formula of Fick method. The result indicated that LVDd and septal e′ were significantly associated with CI at rest (Table 3).

**Table 3:** Independent predictors of cardiac index

Variable	Standard coefficient	*P* value
LVDd	0.430	< 0.001
Septal e′	0.441	0.001

In addition, we analyzed SVI which is an important determinant of CI. Stepwise multiple regression analysis was performed incorporating same independent variables used in CI analysis, except HR. The result indicated that LVDd, septal e′, and BNP were significantly associated with SVI, which was compatible with the result of CI (Table 4).

**Table 4:** Independent predictors of stroke volume index

Variable	Standard coefficient	*P* value
LVDd	0.479	< 0.001
Septal e′	0.383	< 0.001
BNP	− 0.276	0.01

*LVDd* left ventricular end-diastolic dimension; *e′* early diastolic velocity of the septal mitral annulus; *HR* heart rate; *BNP* brain natriuretic peptide

### Exercise capacity and echocardiography

**Fig. 3:**
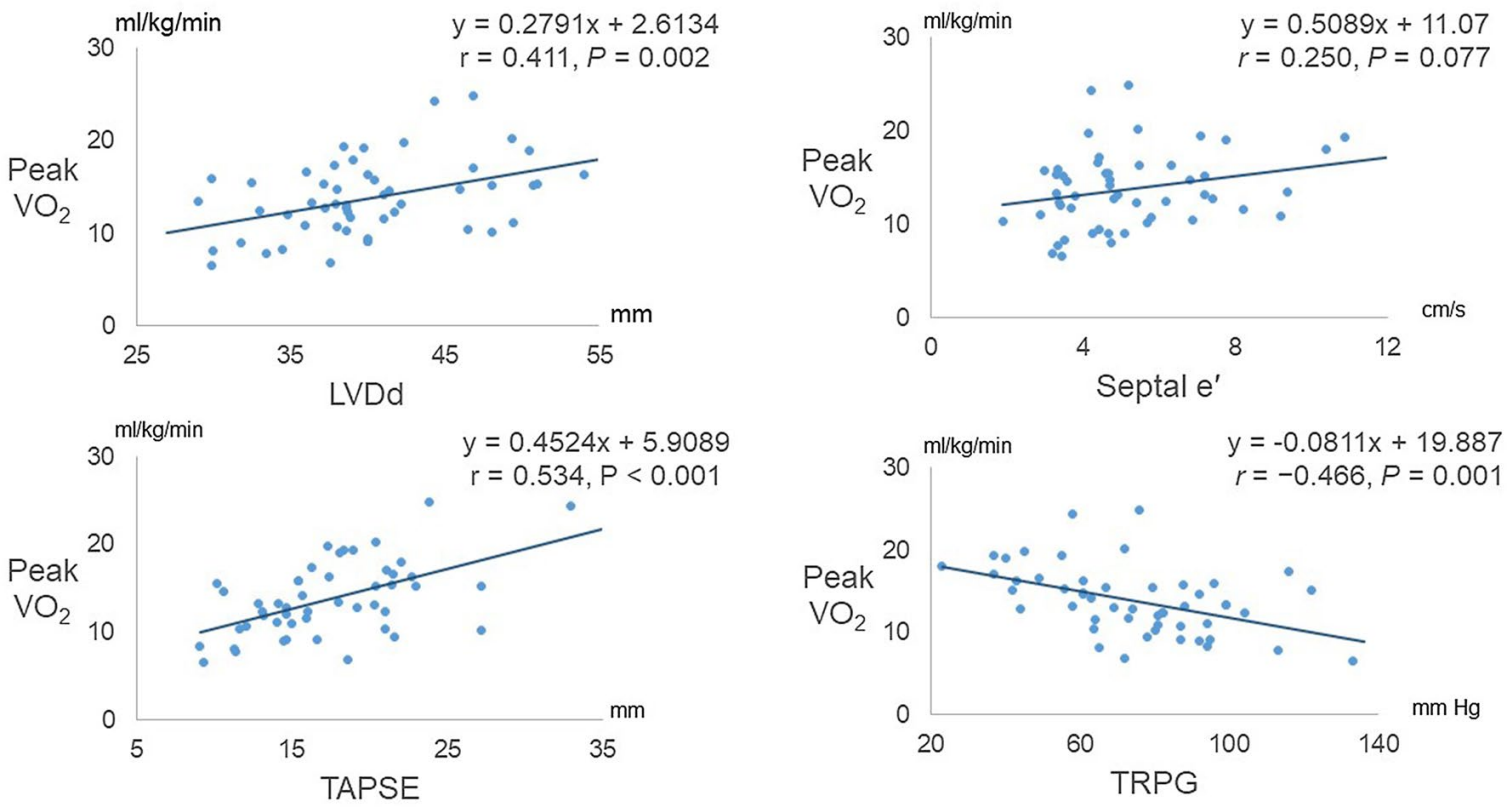
Relation of peak oxygen uptake (VO_2_) to left ventricular end-diastolic dimension (LVDd), early diastolic velocity of the septal mitral annulus (septal e′), tricuspid annular plane systolic excursion (TAPSE), and pressure gradient of tricuspid regurgitation (TRPG)

No complication was observed during CPET. The values for peak VO_2_ indicated severe exercise intolerance in these patients (Table 2). In terms of relationships between parameters of echocardiography and exercise capacity, LVDd and TAPSE were significantly and positively correlated with peak VO_2_, and TRPG was significantly and negatively correlated with peak VO_2_ (Fig. 3).

To identify parameters predictive of peak VO_2_, which is indicative of prognosis in patients with PH, we performed stepwise multiple regression analysis. Among the variables including clinical and laboratory findings, hemodynamic and echocardiographic parameters, the independent variables used in this analysis were World Health Organization functional class (WHO-FC), BNP, heart rate, CI, mean PAP, SvO_2_, LVDd, TAPSE, and TRPG. Among those, TAPSE and SvO_2_ were significantly associated with peak VO_2_ (Table 5).

**Table 5:** Independent predictors of peak oxygen uptake

Variable	Standard coefficient	*P* value
TAPSE	0.544	< 0.001
SvO_2_	0.329	0.005

*TAPSE* tricuspid annular plane systolic excursion; *SvO*_*2*_ mixed venous oxygen saturation

## Discussion

In the present study, LVDd and septal e′ in patients with PAH and CTEPH were significantly associated with CI and SVI. TAPSE was significantly associated with peak VO_2_.

In our present patients with PH, echocardiographic parameters of LV at diastole reflected CI, a key prognostic factor of PAH. As PH progresses, direct or sequential interaction between the RV and LV leads to impaired LV filling. In the direct interaction, the LV cavity is compressed owing to leftward bowing of the ventricular septum, causing impaired LV filling, reduced LV preload, and low cardiac output [21]. In the sequential mechanism, an increased RV afterload decreases RV output, with consequent decreases in LV preload and LV output [27]. These hemodynamic changes due to underfilling of the LV, regardless of the underlying mechanism, result in decreased LV output. Therefore, the echocardiographic parameter LVDd is a good representative of the LV end-diastolic volume, and a decreased e′ indicates an abnormality in LV relaxation.

LVDd was reduced and strongly correlated with SVI and CI in our PAH and CTEPH patients, which is comparable to the study using MRI in patients with PAH [21]. In their study, LV end-diastolic volume was decreased and the LV end-diastolic volume index was strongly correlated with SVI. Eccentricity index measured by echocardiography is used to evaluate LV compression for hemodynamic assessment in PH patients [7]. However, our study indicated that LVDd by itself, which is typically and easily measured during routine echocardiographic examinations, could also be a highly useful parameter that is indicative of CI. Estimated cardiac output can be directly measured by echocardiography. However, LVDd can be more easily measured with less observer bias; thus LVDd also could be a useful marker to assess the severity of patients with PAH and CTEPH. In addition, septal e′ was significantly associated with SVI and CI in our patients with PH, which may indicate abnormal early diastolic underfilling of the LV. In an MRI study of PAH, the filling rate at the time of maximal septal curvature was found to be significantly correlated with the LV end-diastolic volume [21]. In another study, patients with CTEPH who were evaluated before undergoing pulmonary endarterectomy demonstrated LV diastolic dysfunction, which was likely reflective of low LV preloading and underfilling [27]. In our study, mitral E velocity and E/A ratio were decreased to similar levels that were reported in patients with CTEPH by Gurudevan et al. [27]. In addition, the same group reported lateral e′—but not septal e′—improved soon after pulmonary endarterectomy. In our study, whether the reduced septal e′ represents LV underfilling or true LV diastolic dysfunction remains unclear. Nonetheless, our results suggest that evaluation of LV as well as RV functions is important in PH patients.

Peak VO_2_ during exercise is an important prognostic factor in PAH patients [23]. In the present study, low TAPSE, indicating systolic RV dysfunction and low SvO_2_, was significantly associated with low peak VO_2_. Sharma et al. reported a study of dobutamine stress transthoracic echocardiography in patients with PAH, in which both tricuspid annular systolic velocities (s′) measured at rest and after dobutamine stress were strongly correlated with peak VO_2_. [28] Additionally, TAPSE at rest was also significantly correlated with peak VO_2_. These previous results and our results together indicate that RV function is strongly associated with exercise capacity and that assessment of RV systolic function using echocardiography is informative regarding exercise capacity in patients with PAH and CTEPH.

Our study had several limitations. Because it was carried out in the context of current clinical practices, we were unable to perform echocardiography, right heart catheterization, and CPET all on the same day. Right heart catheterization was performed within a median 15 days before and after echocardiography and median 17 days before and after CPET, and each patient received the same drugs with the same dose throughout all examinations. In addition, we evaluated lateral e′, total RV linear measurements, fractional area change, Doppler tissue imaging-derived tricuspid lateral annular systolic velocity wave, RV index of myocardial performance, and longitudinal strain only in limited patients. Therefore, although septal e′ is an established relaxation marker in left-sided heart failure, its usage as a marker for the abnormal LV relaxation, specifically in patients with PAH and CTEPH, remains unclear. Furthermore, because we excluded PH patients with left heart disease, chronic lung disease, or congenital heart disease, future studies are warranted to evaluate whether our results could be applied to PH patients with these comorbidities. Finally, we evaluated patients only at the time of their first admission to our hospital. How each echocardiographic parameter changes as a consequence of patient’s condition remains to be assessed in future.

In conclusion, we demonstrated that, in patients with PAH and CTEPH, LVDd and septal e′ were significantly associated with CI, whereas TAPSE was significantly associated with peak VO_2_. Our results indicate that evaluating both LV and RV functions is important in PAH and CTEPH patients. Furthermore, echocardiography—particularly several simple parameters measured frequently in clinical practice—is useful in predicting CI obtained through invasive techniques at rest and peakVO_2_ during exercise obtained by great physical effort.

## Electronic supplementary material

Below is the link to the electronic supplementary material.

Supplementary material 1 (DOC 33 kb)

Supplementary material 2 (DOC 49 kb)

Supplementary material 3 (DOC 34 kb)
