# Supplementary material for: Left ventricular end-diastolic dimension and septal e′ are predictors of cardiac index at rest, while tricuspid annular plane systolic excursion is a predictor of peak oxygen uptake in patients with pulmonary hypertension
Source: Heart Vessels. 2017 Nov 15;33(5):521–8. doi: 10.1007/s00380-017-1086-0 (PMC5911277; doi:10.1007/s00380-017-1086-0)
Supplement: Supplementary file 1 — Supplementary material 1 (DOC 33 kb) [file 380_2017_1086_MOESM1_ESM.doc]

Supplement 1. Patient characteristics

|  | PAH (n=23) | CTEPH (n=30) |
| --- | --- | --- |
| Age (years) | 47.7 ± 17.2 | 58.3 ±11.7 |
| Male | 5 | 11 |
| Height (cm) | 157.6 ± 10.3 | 160.6 ± 9.7 |
| Weight (kg) | 56.5 ± 15.5 | 64.6 ± 15.8 |
| Body mass index (kg·m^-2^) | 22.7 ± 5.7 | 25.1 ± 5.9 |
| Body surface area (m^2^) | 1.6 ± 0.2 | 1.7 ± 0.2 |
| WHO functional class  1/2/3/4 | 0/13/7/3 | 0/21/8/1 |
| 6MWD (m) | 348.9 ± 111.9 | 407.8 ± 75.0 |
| Monotherapy  ERA  PDE-5 | 3  2 | 1  2 |
| Combination therapy  ERA + PDE-5 | 2 | 2 |
| Plasma BNP (pg/mL) | 131.4 (29.0-335.7) | 80.4 (47.2-217.6) |

Data presented as mean ± SD, median (interquartile range) or n. I/HPAH, idiopathic/heritable pulmonary arterial hypertension; POPH, portopulmonary hypertension; CTD-PAH, connective tissue disease associated pulmonary arterial hypertension; CTEPH, chronic thromboembolic pulmonary hypertension; WHO, world health organization; 6MWD: six minute walk distance; ERA, endothelin receptor antagonist; PDE-5, phosphodiesterase-5 inhibitor; BNP, brain natriuretic peptide.
