# Supplementary material for: Left ventricular end-diastolic dimension and septal e′ are predictors of cardiac index at rest, while tricuspid annular plane systolic excursion is a predictor of peak oxygen uptake in patients with pulmonary hypertension
Source: Heart Vessels. 2017 Nov 15;33(5):521–8. doi: 10.1007/s00380-017-1086-0 (PMC5911277; doi:10.1007/s00380-017-1086-0)
Supplement: Supplementary file 2 — Supplementary material 2 (DOC 49 kb) [file 380_2017_1086_MOESM2_ESM.doc]

Supplement 2. Baseline measured variables

|  | PAH (n=23) | CTEPH (n=30) |
| --- | --- | --- |
| Hemodynamics |  |  |
| PAWP (mm Hg) | 8.8 ± 3.7 | 10.0 ± 3.7 |
| Mean ABP (mm Hg) | 87.5 ± 11.6 | 97.4 ± 13.7 |
| Mean RAP (mm Hg) | 5.8 ± 4.8 | 7.4 ± 3.7 |
| Systolic PAP (mm Hg) | 79.2 ± 19.5 | 80.6 ± 14.0 |
| Diastolic PAP (mm Hg) | 32.0 ± 10.4 | 26.6 ± 6.8 |
| Mean PAP (mm Hg) | 49.7 ± 12.4 | 46.3 ± 8.4 |
| Heart rate (bpm) | 78.3 ± 15.4 | 75.6 ± 15.0 |
| SvO_2_ (%) | 64.2 ± 13.1 | 62.9 ± 6.2 |
| SaO_2_ (%) | 93.2 ± 5.0 | 90.9 ± 4.7 |
| CI (l/min/m^2^) | 2.1 ± 1.0 | 2.0 ± 0.4 |
| SVI (l/m^2^) | 26.6 ± 14.5 | 28.0 ± 7.3 |
| PVR (Wood Unit) | 14.8 ± 8.8 | 11.0 ± 4.0 |
| Cardiopulmonary exercise test |  |  |
| Peak VO_2_ (ml/kg/min) | 13.0 ± 4.1 | 14.4 ± 4.0 |
| O_2_ pulse | 5.0 ± 3.1 | 6.9 ± 2.0 |
| VE/VCO_2_ slope | 50.7 ± 21.7 | 54.6 ± 14.0 |
| peak RER | 1.0 ± 0.3 | 1.0 ± 0.1 |
| Echocardiography |  |  |
| LVDd (mm) | 38.0 ± 6.5 | 41.7 ± 5.1 |
| LVDs (mm) | 24.3 ± 4.8 | 26.0 ± 4.2 |
| IVSd (mm) | 8.2 ± 1.5 | 8.4 ± 1.2 |
| LVPWd (mm) | 8.3 ± 1.5 | 8.5 ± 1.2 |
| LAD (mm) | 31.7 ± 7.3 | 32.9 ± 4.6 |
| TRPG (mm Hg) | 73.5 ± 26.5 | 72.0 ± 24.7 |
| TR grade | 2.7 ± 1.2 | 2.4 ± 0.9 |
| TAPSE (mm) | 15.9 ± 4.5 | 17.2 ± 6.7 |
| E velocity (cm/s) | 58.2 ± 21.6 | 55.7 ± 17.1 |
| A velocity (cm/s) | 63.8 ± 18.2 | 73.7 ± 16.9 |
| E/A | 1.0 ± 0.6 | 0.7 ± 0.2 |
| DcT (ms) | 210.7 ± 83.1 | 236.3 ± 79.8 |
| Septal e′ (cm/s) | 5.5 ± 2.5 | 4.7 ± 1.7 |
| E/e′ | 11.0 ± 8.0 | 12.0 ± 4.8 |

Data presented as mean ± SD, median (interquartile range) or n. PAWP, pulmonary artery wedge pressure; ABP, arterial blood pressure; RAP, right atrial pressure; PAP, pulmonary artery pressure; SvO_2_, mixed venous oxygen saturation; SaO_2_, arterial oxygen saturation; CI, cardiac index; SVI, stroke volume index; PVR, pulmonary vascular resistance; HR, heart rate; VO_2_, oxygen uptake; VE, minute ventilation; VCO_2_, carbon dioxide production; RER, respiratory exchange ratio; LVDd, left ventricular end-diastolic dimension; LVSd, left ventricular end-systolic dimension; IVSd, interventricular septal distance; LVPWd, left ventricular posterial wall distance; LAD, left atrial dimension; TRPG, pressure gradient of tricuspid regurgitation; TR, tricuspid regurgitation; TAPSE, tricuspid annular plane systolic excursion; DcT, deceleration time; e′, early diastolic velocity of the septal mitral annulus.
