# Supplementary material for: Left ventricular end-diastolic dimension and septal e′ are predictors of cardiac index at rest, while tricuspid annular plane systolic excursion is a predictor of peak oxygen uptake in patients with pulmonary hypertension
Source: Heart Vessels. 2017 Nov 15;33(5):521–8. doi: 10.1007/s00380-017-1086-0 (PMC5911277; doi:10.1007/s00380-017-1086-0)
Supplement: Supplementary file 3 — Supplementary material 3 (DOC 34 kb) [file 380_2017_1086_MOESM3_ESM.doc]

Supplement 3.

Correlation between echocardiographic parameters and hemodynamic indices or peak oxygen uptake

| ***r*** | Mean RAP | Mean PAP | CI | SVI | PVR | SvO2 | Peak VO2 |
| --- | --- | --- | --- | --- | --- | --- | --- |
| LVDd | 0.034 | −0.428** | 0.477*** | 0.571*** | −0.588*** | 0.333* | 0.411** |
| LVDs | 0.025 | −0.414* | 0.370** | 0.415** | −0.480*** | 0.174 | 0.244 |
| LAD | 0.140 | −0.284* | 0.053 | 0.156 | −0.330* | 0.03 | −0.084 |
| TAPSE | 0.087 | −0.400** | 0.406** | 0.484*** | −0.550*** | 0.205 | 0.534*** |
| TRPG | 0.145 | 0.486*** | −0.455** | −0.431** | 0.486*** | −0.308* | −0.466** |
| E | 0.186 | −0.473*** | 0.295* | 0.301* | −0.408** | 0.132 | 0.188 |
| Septal e′ | −0.148* | −0.239 | 0.463** | 0.507*** | −0.386** | 0.338* | 0.250 |
| E/A | 0.130 | −0.187 | 0.220 | 0.288* | −0.081 | 0.073 | 0.337* |
| E/e′ | 0.304* | −0.117 | −0.180 | −0.207 | −0.074 | −0.178 | −0.027 |

RAP, right atrial pressure; PAP, pulmonary artery pressure; CI, cardiac index; PVR, pulmonary vascular resistance; SvO2, mixed venous oxygen saturation; VO2, oxygen uptake; LVDd, left ventricular end-diastolic dimension; LVSd, left ventricular end-systolic dimension; LAD, left atrial dimesion; TAPSE, tricuspid annular plane systolic excursion; TRPG, pressure gradient of tricuspid regurgitation; e′, early diastolic velocity of the mitral annulus; *, *P* < 0.05; **, *P* < 0.01; ***, *P* < 0.001.
